# Primary essential cutis verticis gyrata

**DOI:** 10.1590/0100-3984.2017.0218

**Published:** 2019

**Authors:** Isa Félix Adôrno, Rômulo Florêncio Tristão Santos, Thiago Franchi Nunes, Gabriel Barbosa Sandim, Edson Marchiori

**Affiliations:** 1 Universidade Federal de Mato Grosso do Sul (UFMS), Campo Grande, MS, Brazil.; 2 Universidade Federal do Rio de Janeiro (UFRJ), Rio de Janeiro, RJ, Brazil.

**Figure 1:**
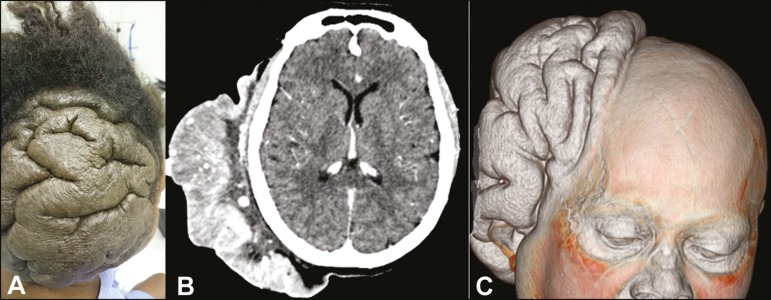
**A:** Photograph of the occipital region, showing a cerebriform cutaneous mass. **B:** Contrast-enhanced axial computed tomography of the skull, showing a lesion involving the subcutaneous tissue of the right parieto-occipital region, with no signs of communication with the brain. **C:** Three-dimensional reconstruction providing a better view of the lesion and of its relationship with the cranial vault.

Dear Editor,

A 53-year-old woman was admitted to the emergency room with a three-day history of self-reported fever and diffuse headache. She reported no history of surgical interventions. On physical examination, her overall health status was satisfactory. However, a cutaneous mass, rich in sulci but without secretions, was observed in the right parietal region (Figure 1A). Computed tomography of the skull showed right-sided cutaneous thickening in the parietal, temporal, and occipital regions, with diffuse microcalcifications, mimicking the appearance of cerebral gyri. The cranial vault and cerebral parenchyma were unaffected (Figure 1B). Three-dimensional reconstruction provided a better view of the lesion and of its relationship with the cranial vault (Figure 1C). Collectively, those findings were consistent with a diagnosis of cutis verticis gyrata (CVG). Local scalp hygiene resulted in clinical improvement. The patient was discharged to outpatient treatment by the dermatology department of our institution.

CVG is a disease characterized by excessive growth of the skin of the scalp, resulting in the formation of sulci and gyri that resemble those of the cerebral cortex. The etiology of CVG is unknown. It is categorized as primary essential, primary non-essential, or secondary^(^^1^^,^^2^^)^.

The primary non-essential form, which accounts for 0.5% of cases, is associated with neurological manifestations such as microcephaly, intellectual disability, cerebral palsy, and epilepsy, as well as ophthalmological manifestations such as cataracts and blindness^(^^1^^,^^3^^)^. The primary essential form is not associated with neurological or ophthalmological alterations, presenting only as scalp folds, which mimic the cerebral gyri, and predominantly affects men; it typically appears during or after puberty, 90% of patients being diagnosed after 30 years of age^(^^1^^,^^3^^,^^4^^)^.

The secondary form, which can occur at any age, affects men and women with similar frequency; the clinical presentation varies depending on the underlying cause, such causes including cerebriform intradermal nevus, inflammatory dermatoses, endocrine diseases, and genetic syndromes^(^^2^^,^^5^^)^. Typically, the scalp folds and furrows seen in CVG show a disordered pattern, with an asymmetric distribution.

An appropriate investigation includes histopathological analysis to determine the etiology. Although the affected area is asymptomatic, there can be accumulation of secretions, causing odor and itching; therefore, good scalp hygiene is important for symptom relief. When secondary to other etiologies, CVG usually regresses after treatment of the underlying disease, although surgical excision may be necessary in this or any of the forms of presentation^(^^1^^,^^4^^,^^6^^)^.
